# Hepatoprotective Effects of Steamed and Freeze-Dried Mature Silkworm Larval Powder against Ethanol-Induced Fatty Liver Disease in Rats

**DOI:** 10.3390/foods9030285

**Published:** 2020-03-04

**Authors:** Da-Young Lee, Kyung-Sook Hong, Moon-Young Song, Sun-Mi Yun, Sang-Deok Ji, Jong-Gon Son, Eun-Hee Kim

**Affiliations:** 1 College of Pharmacy and Institute of Pharmaceutical Sciences, CHA University, Seongnam 13488, Korea; angela8804@naver.com (D.-Y.L.); sukihong83@gmail.com (K.-S.H.); wso219@naver.com (M.-Y.S.); sun21mi@naver.com (S.-M.Y.); 2 Department of Agricultural Biology, National Institute of Agricultural Science, Rural Development Administration, Wanju 55365, Korea; ji35879@daum.net (S.-D.J.); sonjg@korea.kr (J.-G.S.)

**Keywords:** steamed and freeze-dried mature silkworm larval powder, alcoholic fatty liver, ethanol, lipogenesis, fatty acid oxidation, Sprague-Dawley rats

## Abstract

Silkworm, *Bombyx mori*, contains high amounts of beneficial nutrients, including amino acids, proteins, essential minerals, and omega-3 fatty acids. We have previously reported a technique for producing steamed and freeze-dried mature silkworm larval powder (SMSP), which makes it easier to digest mature silkworm. In this study, we investigated the preventive effects of SMSP on alcoholic fatty liver disease and elucidated its mechanism of action. Male Sprague-Dawley rats treated with SMSP (50 mg/kg) or normal diet (AIN-76A) were administered 25% ethanol (3 g/kg body weight) by oral gavage for 4 weeks. SMSP administration for 4 weeks significantly decreased hepatic fat accumulation in ethanol-treated rats by modulating lipogenesis and fatty acid oxidation-related molecules such as sirtuin 1, AMP-activated protein kinase, and acetyl-CoA carboxylase 1. Moreover, SMSP administration significantly diminished the levels of triglyceride in liver tissues by as much as 35%, as well as lowering the serum levels of triglyceride, gamma glutamyl transpeptidase, alanine transaminase, and aspartate aminotransferase in ethanol-treated rats. SMSP supplementation also decreased the pro-inflammatory tumor necrosis factor-alpha and interleukin 1 beta levels and cytochrome P450 2E1 generating oxidative stress. These results suggest that SMSP administration may be possible for the prevention of alcoholic liver disease.

## 1. Introduction

Alcohol-related liver disorder is one of the most common diseases in the world [1]. Chronic alcohol consumption leads to liver diseases including hepatic steatosis, hepatocellular injury, and inflammation, and can cause liver fibrosis, cirrhosis, and hepatocellular carcinoma [2,3]. In particular, as one of the most common forms of alcoholic liver disease, hepatic steatosis is generally considered to be a benign and reversible process that can progress to a more serious condition [4,5]. AMP-activated protein kinase (AMPK) is known to be a major factor of energy metabolism and is involved in lipid metabolism [6]. When AMPK activity is inhibited by several causes, lipogenic transcription factors increase, resulting in fat generation [7]. In addition, AMPK phosphorylates the serine residues of acetyl CoA carboxylase 1 (ACC1), activating catabolic pathways, including fatty acid oxidation [8]. Thus, the regulation of lipogenesis and fatty acid oxidation through AMPK signaling is a therapeutic mechanism for alcoholic steatosis.

After drinking, alcohol is metabolized by cytochrome P450 2E1 (CYP2E1), an enzyme mainly expressed in the liver [3]. Increased CYP2E1 by alcohol consumption causes oxidative stress and inflammation in the kupffer cells, resulting in liver damage [9,10]. In the liver, kupffer cells play a key role in an innate immune system and they accelerate the secretion of pro-inflammatory cytokines, such as tumor necrosis factor-alpha (TNF-α), interleukin (IL)-1β, and IL-6 [11]. Recently, it has been found that these cytokines induce the imbalance of lipid metabolism in the liver and cause the more serious liver diseases [12]. Limuro et al. reported that inhibition of TNF-α inhibited fatty liver in alcohol-treated rats [13]. Moreover, hepatic injury and steatosis induced by alcohol were alleviated in TNF-α knockout mice [13]. Other cytokines, such as IL-1β and IL-6, have been also reported to interfere with lipid metabolism in the liver [14]. Therefore, suppression of the pro-inflammatory cytokines production might have a therapeutic effect against hepatic inflammation and steatosis induced by alcohol consumption.

Recently, there has been interest in edible insects, and they have been highlighted as a future food [15]. The silkworm, *Bombyx mori*, contains high amounts of protein, omega-3 fatty acids, vitamins, and minerals [16]. Silkworms are traditionally used for the treatment of spasms, phlegm, and flatulence [15]. In addition, the silkworm has recently received scientific attention and several studies have reported its health advantages against liver injury [16], Parkinson’s disease [17], and diabetic hyperglycemia [18]. However, after the third day of the fifth instar, the protein composition of silkworm glands rapidly increases, hence, there is a problem insofar as the biological activity is lowered [19]. In addition, dried or refrigerated silkworms are difficult to consume as food because the silk protein is denatured to a high strength [19]. Therefore, Ji et al. have developed a new method of processing silkworms into an easy-to-eat form, in which the mature silkworms are steamed for 130 min at 100 °C before lyophilizing and grinding [19]. We previously reported that steamed and freeze-dried mature silkworm larval powder (SMSP, 0.1, 1, and 10 g/kg) alleviated fatty liver disease in rats treated with alcohol [20]. However, in terms of human dosage, the dose of SMSP reported in the previous studies is burdensome to consume large amounts of silkworm powder per day. In the present study, we aimed to determine whether low dosage of SMSP attenuates hepatic steatosis in rats with ethanol-induced fatty liver and to explore the potential molecular mechanisms of the health benefits of SMSP, focusing on the gene expression involved in lipid metabolism and inflammation in liver.

## 2. Materials and Methods

### 2.1. Preparation of Steamed Mature Silkworm Larval Powder

SMSP was made as previously described [19]. Briefly, live mature larvae of *Bombyx mori*, 7th day of 5th instar silkworms, were immediately steamed for 130 min at 100 °C using an electric pressure-free cooking machine (KumSeong Ltd., Boocheon, Korea) and freeze-dried using freeze-drier (FDT-8612, Operon Ltd., Kimpo, Korea) for 24 h. Silkworms were then grinded using a disk mill (Disk Mill01, Korean Pulverizing Machinery Co. Ltd., Incheon, Korea) and a hammer mill (HM001, Korean Pulverizing Machinery Co. Ltd., Incheon, Korea). And the SMSP was stored at −50 °C and then used for oral gavage for rats.

### 2.2. Animal and Experimental Design

All animal experiment procedures were conducted in accordance with the guidelines and approval of the Institutional Animal Care and Use Committees (IACUC) of the CHA University (reference number: 180002). Three-week-old male Sprague-Dawley (SD) rats weighing 120–130 g were purchased from Orient bio (Seoul, Korea). All animals were housed in a standardized laboratory environment with a 12 h light/dark cycle at constant temperature of 24 °C. After a week of acclimatization, all animals randomly divided into 3 groups (*n* = 8). The normal group was fed with rodent chow diet (Haran 2018s) and orally received 0.2 mL of distilled water. The ethanol-treated group was fed with rodent chow diet and orally received 25% ethanol (3 g/kg body weight) once a day for four weeks. The SMSP-treated group was also fed rodent chow diet and orally received 25% ethanol and SMSP (50 mg/kg body weight) once a day for four weeks. Rats were monitored daily for body weight change for the experimental period. At the end of the experiment, all rats were sacrificed at 4 weeks by carbon dioxide anesthesia. Blood was drawn from the abdominal aorta into a heparin tube. Serum was subsequently obtained by centrifuging the blood at 3000 rpm for 15 min at 4 °C. The livers were excised, rinsed with PBS, and weighed, and the portion of liver was fixed in 10% formalin. Serum and liver samples were stored at −80 °C until analysis.

### 2.3. Serum Analysis

The serum levels of triglyceride, gamma glutamyl transpeptidase (GGT), alanine aminotransferase (ALT), and aspartate aminotransferase (AST) were analyzed using Hitachi automatic analyzer 7600-210 (Hitachi High-Technologies Corporation, Tokyo, Japan). The serum concentration of TNF-α and IL-1β were measured by using a commercial rat ELISA kit (R&D Systems, Minneapolis, MN, USA) following manufacturer’s instruction.

### 2.4. Hepatic Triglyceride Analysis

For the measurement of triglyceride levels in liver tissues, we used the commercial triglyceride assay kit (AB 65336, Abcam, Cambridge, UK). The liver triglyceride was determined as following the manufacturer’s instruction.

### 2.5. Histological Analysis in the Liver

Formalin-fixed liver samples were embedded in paraffin, sliced at 5 µm, followed by sectioning and hematoxylin and eosin (H&E) staining by standard procedures. Histopathological scoring was assessed by an experienced pathologist, who was blinded to the treatment groups. Levels of fatty infiltration and steatosis were graded as 0 point for no hepatocytes affected, 0.5 point for slightly affected (0–5%), 1 point for mildly (5–20%), 2 points for moderately (20–50%), and 3 points for severely (>50%) [21].

### 2.6. RNA Isolation and Gene Expression Analysis

Total mRNA was isolated from the rat livers using Trizol reagent (Invitrogen, Carlsbad, CA, USA) and cDNA was synthesized using Labopass cDNA synthesis kit (Cosmogenetech Co., Ltd., Seoul, Korea) according to the manufacturer’s instructions. The mRNA levels were analyzed by quantitative real-time PCR (qRT-PCR). The qRT-PCR was assessed as previously reported [22] and was performed on a ViiA^TM^ 7 real-time PCR system (Life Technologies Corporation, Carlsbad, CA, USA) using Luna universal qPCR master mix (New England Biolabs, Beverly, MA, USA). 18S ribosomal RNA (18s rRNA) were used as an internal control. The primer sets for qRT-PCR and RT-PCR are listed in Table 1.

### 2.7. Western Blotting

Liver tissues were grinded with cell lysis buffer containing protease inhibitor (Roche Applied Science, Mannheim, Germany) and samples were incubated on ice with frequent vortexing for 10 min and centrifuged for 15 min at 13,000 rpm. The protein concentration of each supernatant was quantified using Pierce^TM^ BCA protein assay kit (Thermo Fisher Scientific, Waltham, MA, USA) in accordance with the manufacturer’s instructions. The proteins were loaded onto sodium dodecyl sulfate polyacrylamide gel electrophoresis (SDS-PAGE), and transferred to polyvinylidene fluoride membranes (Millipore, Burlington, MA, USA). After transfer, membranes were blocked with bovine serum albumin (BSA) solution and probed with the specified primary antibodies (diluted 1:1000) overnight at 4 °C. The membranes were washed and incubated with the appropriate secondary antibodies for 40 min. The blots were then developed using an enhanced chemiluminescence system (Thermo Fisher Scientific, Waltham, MA, USA). Antibodies for p-AMPK, AMPK, peroxisome proliferator-activated receptor gamma (PPARγ) and β-actin were purchased from Santa Cruz Biotechnology (Santa Cruz, CA, USA). Antibodies for ACC1 and p-ACC1 were purchased from Cell Signaling Technology (Danvers, MA, USA).

### 2.8. Statistical Analysis

All data are expressed as means ± standard deviation (SD). Statistical analysis was performed using one-way analysis of variance (ANOVA). Statistical significance was accepted at *p* < 0.05. 

## 3. Results

### 3.1. SMSP Supplementation Alleviates Hepatic Steatosis in Ethanol-Treated Rats

**Figure 1:**
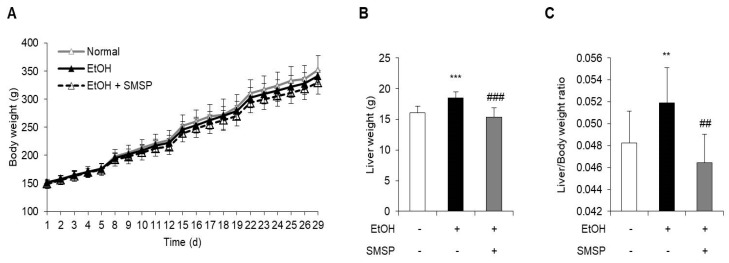
Effect of steamed and freeze-dried mature silkworm larval powder (SMSP) on body weight and liver weight in rats treated with ethanol (EtOH) for 4 weeks. (**A**) Body weight changes, (**B**) liver weight at the end of experiment, and (**C**) liver/body weight ratio were measured. The data represent mean ± SD (*n* = 8); ** *p* < 0.01 and *** *p* < 0.001 vs. normal group; ## *p* < 0.01 and ### *p* < 0.001 vs. EtOH group.

**Figure 2:**
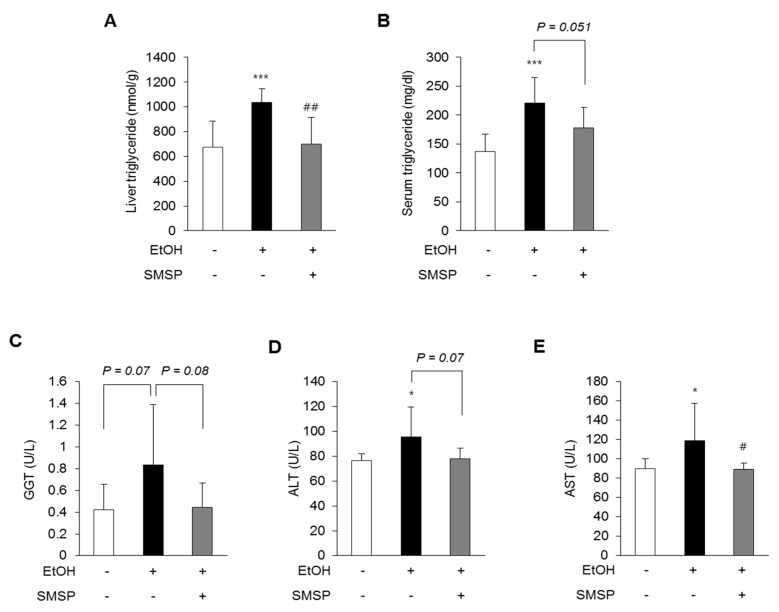
SMSP administration alleviates hepatic steatosis in EtOH-treated rats. (**A**) Hepatic triglyceride and serum levels of (**B**) triglyceride, (**C**) gamma-glutamyl transpeptidase (GGT), (**D**) alanine aminotransferase (ALT), (**E**) aspartate aminotransferase (AST) were measured. The data represent mean ± SD (*n* = 8); * *p* < 0.05 and *** *p* < 0.001 vs. normal group; # *p* < 0.05 and ## *p* < 0.01 vs. EtOH group.

**Figure 3:**
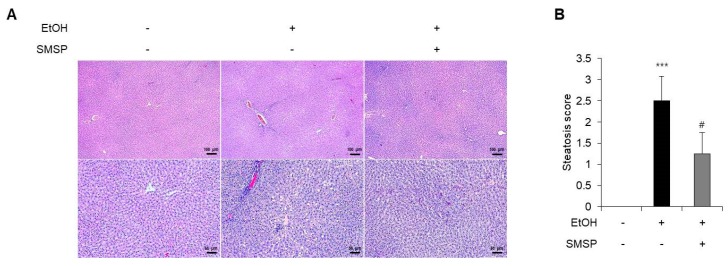
SMSP administration attenuates hepatic lipid accumulation in EtOH-treated rats. (**A**) Histopathological sections of liver were stained with H&E (magnification, ×40 and 100). (**B**) Hepatic steatosis scores were quantified from H&E-stained sections. The data represent mean ± SD (*n* = 4); *** *p* < 0.001 vs. normal group; # *p* < 0.05 vs. EtOH group.

To examine the hepatoprotective role of SMSP, we used an ethanol-induced hepatic steatosis rat model. The ethanol (3 g/kg) and SMSP (50 mg/kg) were orally injected into SD rats for 4 weeks. SMSP administration for 4 weeks significantly reduced the total liver weight compared with ethanol-treated rats without affecting body weight change (Figure 1A,B). The SMSP group, as compared with the ethanol group, had a reduction in liver weight to body weight ratio (Figure 1C). Ethanol treatment for 4 weeks successfully induced fatty liver and liver injury in rats, which were manifested by significant increases in serum triglyceride, GGT, ALT, and AST activities compared with those of normal diet-fed rats (Figure 2B–E). In the meantime, SMSP significantly reversed the ethanol-treated hepatic accumulation of triglyceride by as much as 35% (Figure 2A), as well as lowering the serum triglyceride, GGT, ALT, and AST activities by 15%, 41%, 8.3%, and 9.4%, respectively (Figure 2B–E). In addition, steatosis scores were evaluated in H&E staining images of liver tissues from all groups. As a result, hepatic lipid accumulation was remarkably increased in ethanol-treated rats (Figure 3A). As shown in Figure 3B, the ethanol-induced elevation of the steatosis score was significantly normalized in the SMSP-treated rats.

### 3.2. SMSP Supplementation Attenuates Inflammation in Ethanol-Treated Rats

**Figure 4:**
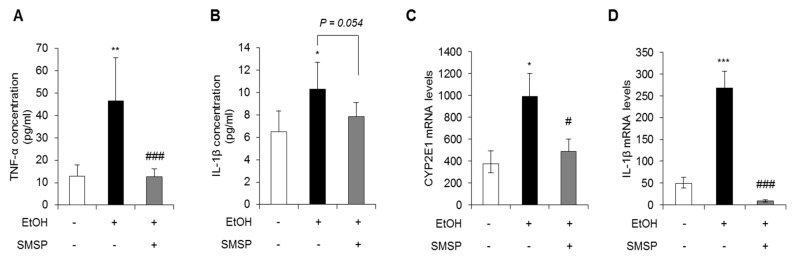
SMSP administration attenuates inflammation in EtOH-treated rats. Serum levels of (**A**) tumor necrosis factor-alpha (TNF-α) and (**B**) interleukin 1 beta (IL-1β) were measured by ELISA. The mRNA expressions of (**C**) cytochrome P450 2E1 (CYP2E1) and (**D**) IL-1β were detected by qRT-PCR in liver tissues from each group. The data represent mean ± SD (*n* = 4); * *p* < 0.05, ** *p* < 0.01, and *** *p* < 0.001 vs. normal group; # *p* < 0.05 and ### *p* < 0.001 vs. EtOH group.

Chronic alcohol consumption leads to liver injury, fat metabolism, inflammation, and hepatocellular carcinogenesis [3]. In addition, chronic alcohol consumption induces the production of reactive oxygen species (ROS), regulated by cytochrome P450 2E1 (CYP2E1), that lead to the production of reactive aldehydes with potent pro-inflammatory properties [11]. Based on this rationale, we analyzed the serum levels of TNF-α and IL-1β. As shown in Figure 4A,B, the TNF-α and IL-1β were significantly increased in ethanol-treated rats, while SMSP treatment attenuated the serum levels of TNF-α and IL-1β. Moreover, the hepatic mRNA expression of the CYP2E1 and IL-1β were significantly decreased by SMSP administration (Figure 4C,D). These results indicate that SMSP supplementation ameliorates liver inflammation induced by ethanol.

### 3.3. SMSP Supplementation Improves Lipid Metabolism in Ethanol-Treated Rats

**Figure 5:**
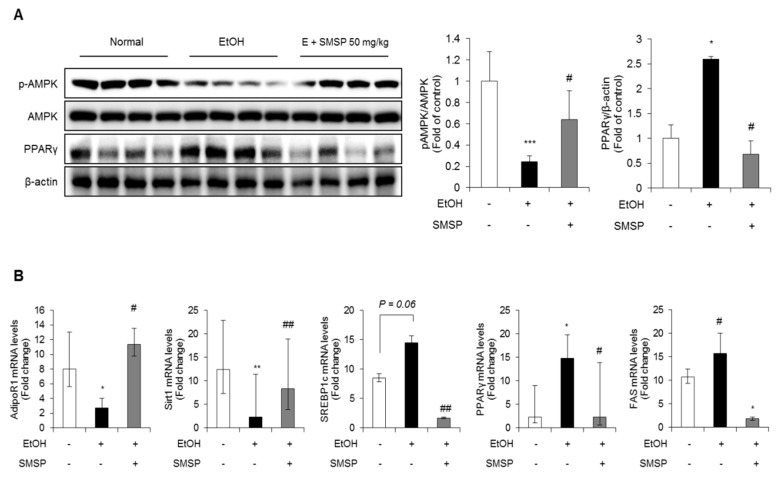
SMSP administration improves lipid metabolism in ethanol-treated rats. (**A**) The expression of phosphorylation of AMP-activated protein kinase (p-AMPK), AMPK and peroxisome proliferator-activated receptor gamma (PPARγ) in the liver tissues were determined by Western blotting and normalized to that of β-actin. (**B**) The mRNA expression of adiponectin receptor 1 (AdipoR1), sirtuin 1 (Sirt1), sterol regulatory element-binding proteins (SREBP1c), PPARγ and fatty acid synthase (FAS) in liver tissues were determined by qRT-PCR and normalized to that of 18s rRNA. The data represent mean ± SD (*n* = 4); * *p* < 0.05, ** *p* < 0.01, and *** *p* < 0.001 vs. normal group; # *p* < 0.05 and ## *p* < 0.01 vs. EtOH group.

To examine the underlying molecular mechanism, we examined the effect of SMSP supplementation on hepatic lipid metabolism such as lipogenesis and free fatty acid (FFA) oxidation. AMPK is one of the key activators of the lipogenesis and FFA oxidation catabolism [23]; we tested whether prolonged SMSP supplementation could modulate the AMPK pathways in the liver of alcohol-treated rats. Western blot results showed that SMSP administration significantly increased the phosphorylation of AMPK reduced by ethanol treatment. However, PPARγ, the well-known lipogenic factor, significantly decreased in the livers of SMSP-fed rats (Figure 5A). The expression of lipogenic nuclear transcription factors, such as sterol regulatory element-binding protein 1c (SREBP1c), PPARγ, and fatty acid synthase (FAS), were down-regulated in the liver of SMSP-treated rats compared with the ethanol-treated rats (Figure 5B). However, SMSP supplementation restored the mRNA levels of adiponectin receptor 1 (AdipoR1) and sirtuin 1 (Sirt1) in the livers of ethanol-treated rats (Figure 5B). 

**Figure 6:**
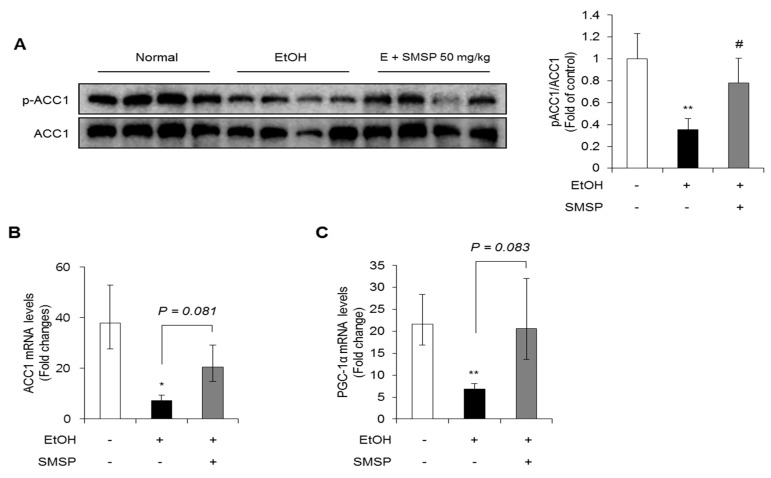
SMSP administration affects the expressions of lipogenesis-related molecules in ethanol-treated rats. (**A**) The phosphorylation of acetyl-CoA carboxylase 1 (ACC1) in the liver tissues was determined by Western blotting and normalized to that of β-actin. The mRNA expression of (**B**) ACC1 and (**C**) peroxisome proliferator-activated receptor gamma coactivator-1 alpha (PGC-1α) in liver tissues was determined by qRT-PCR and normalized to that of 18s rRNA. The data represent mean ± SD (*n* = 4); * *p* < 0.05 and ** *p* < 0.01 vs. normal group; # *p* < 0.05 vs. EtOH group.

The inhibition of ACC1 has been reported to reduce hepatic triglyceride accumulation by decreasing lipogenesis and increasing FFA oxidation [24]. Therefore, we investigated the expression of ACC1 and the phosphorylation. As shown in Figure 6A,B, the phosphorylation of ACC1 was significantly inhibited in ethanol-treated rats while SMSP supplementation recovered the phosphorylation of ACC1. Moreover, the decreased hepatic expressions of ACC1 and peroxisome proliferator-activated receptor-gamma coactivator-1 alpha (PGC-1α) mRNA by ethanol treatment were recovered in SMSP-administered rats, although not statistically significant (Figure 6B,C). These results suggest that SMSP ameliorates EtOH-induced fatty liver through the regulation of lipogenesis and FFA oxidation mediated by AMPK and ACC1.

## 4. Discussion

Insects are used for food and animal feed because they are rich in beneficial ingredients, such as proteins, fats, vitamins, minerals, and fiber [25]. In particular, the silkworm *Bombyx mori* has traditionally been used as food in Asia. Our colleagues have compared the nutrient composition of SMSP with those of freeze-dried mature silkworm powder (FMSP) and freeze-dried 3rd day of 5th instar silkworm powder (FDSP) [16]. A proximal analysis revealed that SMSP shows the highest protein contents compared to FMSP and FDSP. In addition, as shown in Table S1, amino acids such as glycine, alanine, and serine, which are the major components of silk protein are very abundant in SMSP. Moreover, the amount of unsaturated fatty acids is more than twice as high than that of saturated fatty acids in SMSP [16]. In addition, the amount of polyphenol, flavonoids, and minerals were examined. Since the amino acids and n-3 fatty acids have been shown to be effective in preventing metabolic diseases and promoting human health [26,27], in line with this notion, we suggest that the administration of SMSP may have beneficial effects on health based on its nutritional composition. 

In our previous study, we confirmed the hepatoprotective effects of SMSP 0.1, 1, and 10 g/kg in diethylnitrosamine (DEN)-induced acute liver injury rat model and ethanol-induced liver damage rat model [20,28]. We did not show any side effects such as weight change or decreased dietary intake in rats treated with SMSP. In addition, the previous study with DEN-induced hepatocellular carcinoma rat model have shown that the administration of 1 g/kg of SMSP for 16 weeks does not induce any toxicity compared to the normal group [29]. These results demonstrate that the supplementation of 1–10 g/kg rat body weight of SMSP for 4–16 weeks would be safe. However, 1 g/kg of SMSP in rats corresponds to an intake of approximately 9.73 g/60 kg adult/day, when calculated on the basis of normalization to body surface area [30]. Therefore, this study was conducted to investigate the hepatoprotective effect of the lower dose of SMSP, 50 mg/kg rat body weight, corresponding to an intake of 0.487 kg/60kg in human dose. 

Chronic alcohol intake can cause the progress of hepatic steatosis, fibrosis, cirrhosis, and hepatocellular carcinoma (HCC) [17]. Alcohol is a small molecule that can spread easily through the cell membrane [17]. The major enzymes of alcohol metabolism are alcohol dehydrogenase (ADH) and aldehyde dehydrogenase (ALDH) [31]. These enzymes are known as phase 1 xenobiotic metabolize enzymes [31]. ADH rapidly oxidizes alcohol to acetaldehyde, and then ALDH converts acetaldehyde to acetate [32]. This metabolism uses NAD^+^ and is based on CYP2E1. The ADH, CYP2E1, and ALDH are mainly produced in hepatocytes [2]. We previously reported that SMSP supplementation alleviated the serum levels of ADH and ALDH in acute alcohol-induced liver injury rat models [33]. In this study, to explore the protective effect of SMSP in rats, we evaluated the anti-hepatosteatotic effect of SMSP using the fatty liver model induced by ethanol (3 g/kg, daily, 4 weeks). Based on our previous study, this dosage was enough to induce hepatic steatosis in SD rats [20]. We found that rats fed the rodent chow diet and oral gavage of SMSP induced no changes, such as in body weight (Figure 1A), but the mass of liver tissue and liver weight/body weight ratio from rats treated SMSP were 8% and 10% respectively lower than those received ethanol only (Figure 1B,C). In addition, SMSP significantly alleviated the levels of liver triglycerides, serum triglycerides, and liver injury markers, such as GGT, ALT, and AST (Figure 2C–E).

Alcoholic liver disease is characterized by the accumulation of large amounts of lipids in the liver with inflammation [11]. In alcoholic liver diseases, chronic ethanol consumption stimulates Kupffer cells to activation by lipopolysaccharides through diverse signal such as Toll-like receptors [11]. This regulation induces the production of various pro-inflammatory cytokines, such as TNF-α, interferon gamma (IFN-γ), and IL-1β, which are critically regulated hepatic inflammation, steatosis, fibrosis, and HCC [34]. It has been reported that both patient and animal model with alcoholic liver disease, the levels of IL-1β are significantly increased in the liver and the serum [35,36]. The present study showed that SMSP treatment can attenuate the serum levels of TNF-α and IL-1β as well as mRNA levels of IL-1β (Figure 4). These results indicate that SMSP can reduce the production of pro-inflammatory cytokines by the chronic alcohol consumption.

In addition, chronic alcohol intake also induces the oxidative stress that induces lipid peroxidation, intracellular membrane damage, and lead to the production of pro-inflammatory cytokines and pro-fibrotic effects. Oxidative stress and ROS are caused through the CYP2E1 signaling [11]. It has been reported that CYP2E1 knock-out mice exhibit ethanol-induced liver disease [37], and we previously demonstrated that SMSP supplementation restored total antioxidant concentration levels and significantly reduced hepatic malondialdehyde levels [20]. In this study, we also found that rats fed SMSP reduced CYP2E1 mRNA levels even the low dosage of SMSP (Figure 4C). These results suggest that low dosage of SMSP can diminish oxidative stress through inhibition of CYP2E1 induced by ethanol.

The accumulation of triglycerides in hepatocytes causes hepatic steatosis, because of the imbalance between lipogenesis and FFA oxidation. Considerable evidence has reported that adiponectin plays a key role in alcoholic fatty liver in several animal models [38,39,40,41]. Circulating adiponectin binds to adiponectin receptors in the liver and thereby regulates lipid metabolism through AMPK [42]. In hepatocytes, AMPK is a crucial regulator of intracellular energy sensors that has been reported in the regulation of lipid homeostasis [43]. The inactivation of AMPK negatively regulated FFA oxidation through the inhibition of the transcription factors ACC1 and PGC-1α, and increased activation of SREBP1c, a lipogenic transcription factor [44]. Several studies indicate a relationship between SIRT1 and the AMPK signaling pathway [45]. The upregulation of SIRT1 acts on AMPK upstream, suggesting that the regulation of the SIRT1/AMPK signal may act as a key mechanism for lipid homeostasis in liver. In this study, the protein and mRNA levels of genes involved in SIRT1/AMPK-mediated lipogenesis and FFA oxidation were regulated in rats administered SMSP. These results indicate that SMSP improves lipid metabolism in the livers of rats treated with ethanol.

In summary, the present study suggests that the low dose of SMSP (50 mg/kg) effectively reduced the hepatic steatosis through the activation of SIRT1/AMPK-mediated signaling cascades in ethanol-treated rats. Increased hepatic SIRT1 and AMPK activity appears to be associated with these beneficial effects of SMSP. Further studies on the effect of SMSP in vitro and in humans should confirm that SMSP could serve as an effective therapeutic agent in treating human alcoholic fatty liver disease.

## Figures and Tables

**Table 1:** List of primers.

Gene	Forward	Reverse	Size (bp)
CYP2E1	AAA CAG GGT AAT GAG GCC CG	AGG CTG GCC TTT GGT CTT TT	75
IL-1β	CAC CTT CTT TTC CTT CAT CTT TG	GTC GTT GCT TGT CTC TCC TTG TA	240
AdipoR1	AGG TCA AAC GTG ACG GCT C	TTA GGC CTG TCG ACT CTC CA	71
SIRT1	CCC AGA TCC TCA AGC CAT GTT	TTG TGT GTG TGT TTT TCC CCC	120
SREBP1c	CCA TGG ACG AGC TAC CCT TC	GGC ACT GGC TCC TCT TTG AT	382
PPARγ	AGA AGG CTG CAG CGC TAA AT	GGC CTG TTG TAG AGT TGG GT	405
FAS	TCG ACT TCA AAG GAC CCA GC	GGC AAT ACC CGT TCC CTG AA	228
ACC1	CTT GGG GTG ATG CTC CCA TT	GCT GGG CTT AAA CCC CTC AT	116
PGC-1α	TAA ACT GAG CTA CCC TTG GG	CTC GAC ACG GAG AGT TAA AGG AA	89
18s rRNA	GCA ATT ATT CCC CAT GAA CG	GGC CTC ACT AAA CCA TCC AA	111

## Supplementary Materials

The following are available online at https://www.mdpi.com/2304-8158/9/3/285/s1, Table S1: The nutrient composition of SMSP.
