# Supplementary material for: Hepatoprotective Effects of Steamed and Freeze-Dried Mature Silkworm Larval Powder against Ethanol-Induced Fatty Liver Disease in Rats
Source: Foods. 2020 Mar 4;9(3):285. doi: 10.3390/foods9030285 (PMC7142575; doi:10.3390/foods9030285)
Supplement: Supplementary file 1 [file foods-09-00285-s001.pdf]

foods

MDPI

Table S1. The nutrient composition of SMSP.

Nutrient (unit)	SMSP	
Proximal analysis (g/100 g)	Water	2.85
	Crude protein	68.66
	Crude fat	10.99
	Crude ash	3.26
	Crude fiber	1.77
Amino acids (%)	Glycine	12.335
	Alanine	9.778
	Serine	6.757
	Aspartic acid	4.679
	Tyrosine	4.560
	Glutamic acid	3.824
	Valine	2.399
	Lysine	2.193
	Threonine	2.185
	Leucine	1.983
	Arginine	1.958
	Phenylalanine	1.746
	Isoleucine	1.214
	Proline	1.148
	Histidine	1.083
	Methionine	0.644
	Cysteine	0.427
	Tryptophane	0.425
Vitamins	Vitamin A (μg/100 g)	576.80
	Vitamin B1 (mg/100 g)	0.26
	Vitamin B2 (mg/100 g)	3.67
	Vitamin B6 (mg/100 g)	0.35
	Vitamin B12 (μg/100 g)	0.00
	Niacin (mg/100 g)	1.28
	Folic acid (mg/100 g)	0.40
	Biotin (μg/100 g)	0.00
	Vitamin C (mg/100 g)	12.62
Polyphenols (mg/100 g)	Flavonoids	380.16
Minerals (mg/100 g)	Potassium (K)	1045.4
	Phosphorus (P)	688.27
	Sulfur (S)	326.45
	Magnesium (Mg)	199.25
	Calcium (Ca)	198.90
	Sodium (Na)	14.93
	Zinc (Zn)	5.76
	Iron (Fe)	3.17
	Manganese (Mn)	1.60
	Copper (Cu)	1.20
	Chromium (Cr)	0.00
	Lead (Pb)	0.00
	Cadmium (Cd)	0.00
	Mercury (Hg)	0.00
	Arsenic (As)	0.00
Fatty acids (%)	Myristic acid (C14:0)	0.17
	Palmitic acid (C16:0)	24.49
	Stearic acid (C18:0)	8.55

	Oleic acid (C18:1n-9)	31.22
	Palmitoleic acid (C16:1n-7)	0.86
	Linolenic acid (C18:3n-3)	27.86
	Linoleic acid (C18:2n-6)	6.50
	Eicosenoic acid (C20:1n-9)	0.23
	γ-Linoleic acid (C18:3n-6)	0.13
	Vaccenic acid (C18:1n-7)	0.00
	Eicosatrienoic acid (C20:3n-3)	0.00
	Eicosapentaenoic acid (C20:5n-3)	0.00
	Docosatetraenoic acid (C22:4n-6)	0.00
	Docosahexaenoic acid (C22:6n-3)	0.00
	Total	100.00
	▪ Saturated fatty acids	33.21
	▪ Unsaturated fatty acids	66.79
	▪ Mono-unsaturated	32.31
	▪ Poly-unsaturated	34.49
	▪ n-6/n-3	0.24
